# Plasmablastic Lymphoma in an Immunocompetent Patient: A Case Report and Literature Review

**DOI:** 10.7759/cureus.30732

**Published:** 2022-10-26

**Authors:** Mohammed AlSheef, Yacoub Abuzied, Bakhitah Aleid, Noura Shafi, Wafaa Alshakweer, Abdul Rehman Z Zaidi

**Affiliations:** 1 Internal Medicine and Thrombosis, Medical Specialties Department, King Fahad Medical City, Riyadh, SAU; 2 Nursing Department, Rehabilitation Hospital, King Fahad Medical City, Riyadh, SAU; 3 Medical Specialties Department, King Fahad Medical City, Riyadh, SAU; 4 Endocrinology Department, King Fahad Medical City, Riyadh, SAU; 5 Histopathology Department, King Fahad Medical City, Riyadh, SAU; 6 Department of Family and Community Medicine, College of Medicine, Alfaisal University, Riyadh, SAU

**Keywords:** lymphoma, aggressive malignancy, b cell neoplasm, immunocompetent, plasmablastic lymphoma

## Abstract

Plasmablastic lymphoma (PBL) is considered an aggressive rare variant of diffuse large B-cell lymphoma that has a predilection to develop in immunocompromised patients, particularly HIV-positive individuals. This report highlights the development of such a rare and aggressive malignancy in a 55-year-old immunocompetent male. It outlines the atypical clinical presentation and pathological features of this disease entity. Overall prognosis and response to chemotherapy differ in the absence or presence of immunosuppression. This report includes a summary of the epidemiologic, clinical, and immunohistochemical characteristics of PBL based on a comprehensive review of the reported cases occurring in immunocompetent individuals.

## Introduction

Plasmablastic lymphoma (PBL), a previously known variant of diffuse large B-cell lymphoma, is currently recognized by the 2008 World Health Organization (WHO) classification of lymphoma as a distinctive entity of B-cell neoplasm [1,2]. It is composed of diffuse proliferation of large neoplastic cells, most of which resemble B-immunoblasts and have immunophenotype of plasma cells [1,3]. These overlapping features between myeloma and lymphoma that have plasmablastic morphology create a diagnostic challenge for PBL.

PBL is a rare type of lymphoma that has a predilection to affect HIV-positive patients with a high affinity to involve extra-nodal sites, predominantly the oral cavity [3]. Epstein-Barr virus (EBV) also plays a role in the pathogenesis of PBL [4]. Nevertheless, it has been reported that PBL occurs in immunocompetent individuals, particularly the elderly [4]. Regarding the clinical course of PBL in immunocompetent patients, it tends to involve extra-oral sites, with a more aggressive clinical behavior and poor prognosis [5].

For instance, it is estimated to have an overall survival of 19 months despite the use of various chemotherapy regimens [5]. Furthermore, PBL creates a diagnostic challenge for both clinicians and pathologists due to its varying clinical, histological, and immunohistochemical presentations. This report highlights the unusual presentation and pathological features of PBL in an immunocompetent patient.

## Case presentation

A 55-year-old gentleman was diagnosed to have unprovoked right leg deep venous thrombosis (DVT) three months prior to his presentation to the thrombosis clinic which was diagnosed based on Doppler ultrasound findings. For this, he was maintained on a therapeutic dose of rivaroxaban (20 mg daily) and investigated for thrombophilia but all test results were negative. During the current visit, he presented with a one-month history of progressive unilateral lower limb pain and swelling. Otherwise, he was healthy with no chronic medical illness. He gave a history of weight loss, approximately 8 kg over a period of three months, but no history of fever or night sweats.

**Figure 1:**
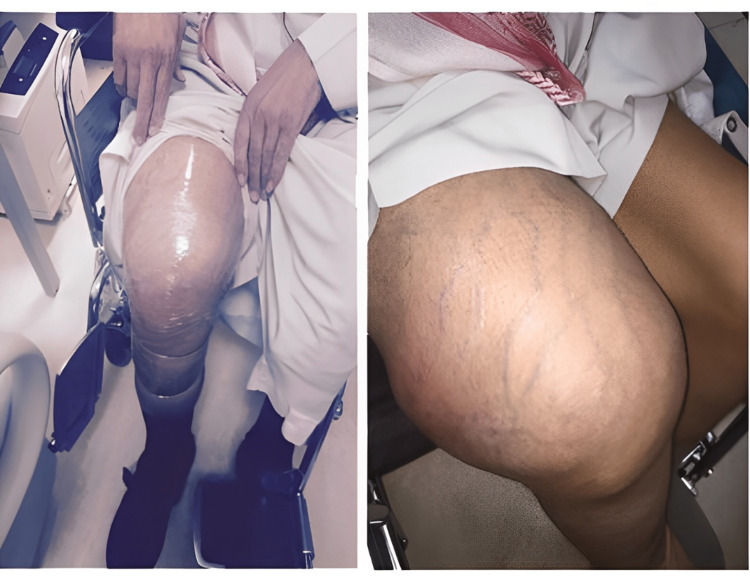
Right thigh swelling with engorged superficial veins.

On physical examination, he had a large right warm and extremely tender middle thigh mass measuring 19 × 18 × 35 cm with overlying erythematous skin and dilated superficial veins. There was lower limb pitting edema; however, peripheral pulses were palpable (Figure 1).

Routine blood examination revealed a white blood cell count of 26.09 × 10^9^/L, neutrophils 88.10%, hemoglobin 10.00 g/dL, platelets 352 × 10^9^/L, plasma lactate dehydrogenase 1,709 U/L, alkaline phosphatase 255, erythrocyte sedimentation rate 118 mm/hour, and C-reactive protein 288 mg/dL. Vascular ultrasound of bilateral lower limb veins showed no evidence of acute DVT but there was a big isoechoic heterogeneous-looking lesion extending over the right thigh until below the knee, and internal vascularity was noted.

**Figure 2:**
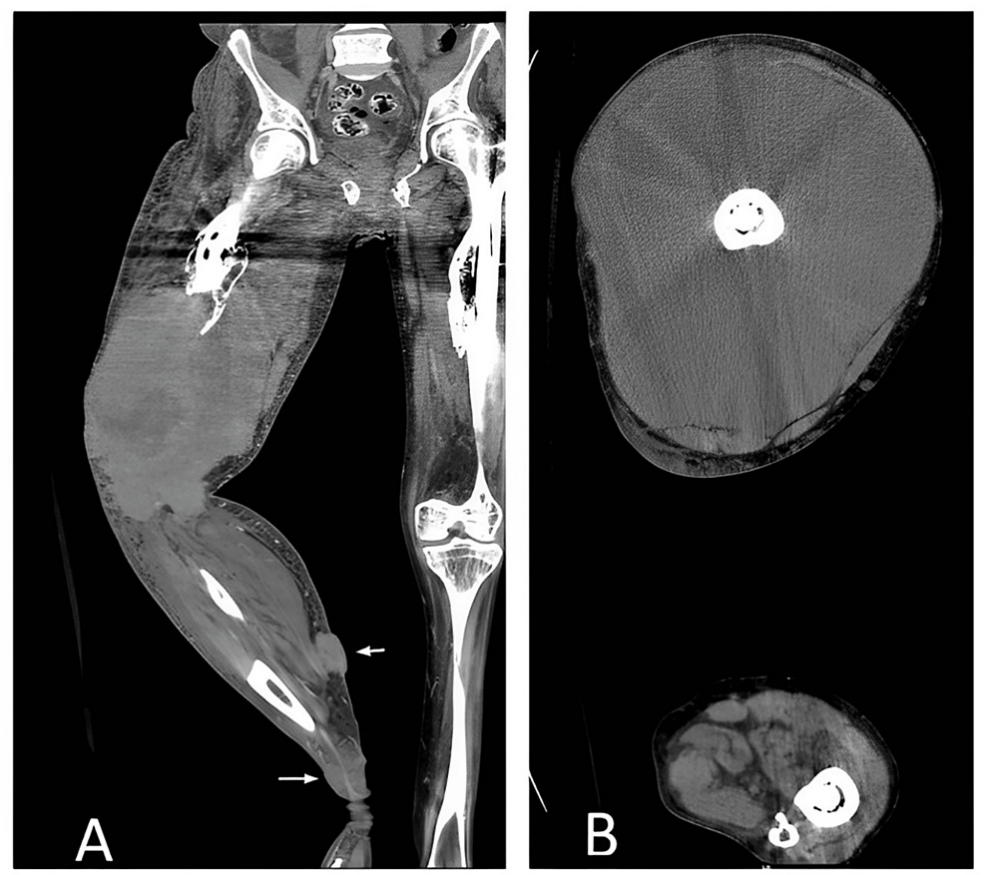
Computed tomography scan of the thigh showing a large heterogeneous soft-tissue mass measuring approximately 19.8 × 18.5 × 35 cm.

Radiographs and computed tomography (CT) scan of the thigh (Figure 2) showed a large heterogeneous soft-tissue mass measuring approximately 19.8 × 18.5 × 35 cm in the transverse, anteroposterior, and cranial-caudal diameter, respectively. The mass was occupying all compartments of the thigh (quadriceps, hamstring, and adductors). The mass had an intra-articular extension to the knee joint. There was bone destruction involving the distal femoral condyles. There were multiple subcutaneous lesions/nodules in the leg.

**Figure 3:**
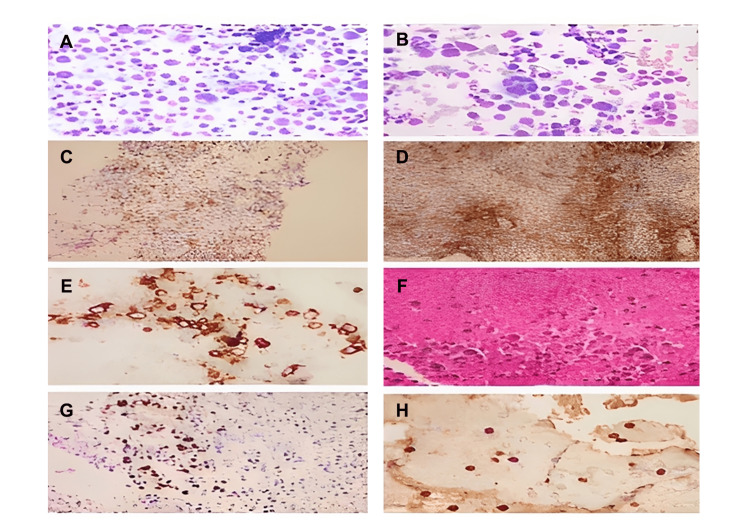
Immunohistopathology findings. (A) Monster cell with emperipolesis among numerous cells with perinuclear huff (immature plasma cells). (B) Immature plasma cells. (C) CD45 positive reaction on the cell block. (D) CD30 focal positivity on the tissue section. (E) CD138 positive reaction on the cell block. (F) Cell block: immature cells admixed with neutrophils, frequent mitotic figures, and numerous necrotic cells. (G) Epstein-Barr virus-in situ hybridization is positive on the tissue. (H) Myeloperoxidase focal positivity.

An ultrasound-guided fine needle biopsy was done which showed pleomorphic large cells with prominent nucleoli and intact membranes. Therefore, anaplastic lymphoma versus granulocytic sarcoma was suspected and further immunohistochemical studies were requested (Figure 3). The neoplastic cells showed focal CD45 and CD79a positivity, diffuse CD138, EBV-ISH, CD45RO, CD30, BCL2, and MUM-1.

Cells were negative for CD19, CD21, CD23, CD56, MPO CD20, PAX5, CD2, CD3, CD4, CD5, CD7, CD8, CD10, TDT, Synaptophysin, ALK-1, GRANZ-B, CK, EMA, S100, and BCL-6. The study was inconclusive for CD99. The cells also showed surface Kappa light chain restriction.

A bone marrow biopsy revealed hypercellularity (80%) with no evidence of lymphoma involvement. Serological testing demonstrated negativity for HIV 1 and 2, hepatitis B virus (HBV) surface antigen, and anti-hepatitis C virus. CT of the chest, abdomen, and pelvis showed no evidence of intra-abdominal hematoma, suspicious bony lesion, suspicious pulmonary nodules, or lymphadenopathy apart from multiple sub-centimetric retroperitoneal and mesenteric lymph nodes.

Based on the previous findings, a final diagnosis of PBL stage II was determined, with a single extra-nodal lesion represented by a thigh mass and two nodal groups in the same diaphragm (retroperitoneal and mesenteric lymph nodes). His hospital course was complicated by disseminated intravascular coagulation, tumor lysis syndrome, and acute kidney injury that required renal replacement therapy.

The case was discussed in the tumor board, and he was started on vincristine and methylprednisolone with a plan to start standard EPOCH (rituximab, etoposide, prednisolone, oncovin, cyclophosphamide, and hydroxydaunorubicin) chemotherapy regimen once he was clinically stable and able to tolerate the treatment. The patient continued to deteriorate clinically and developed multiorgan failure and unfortunately died.

## Discussion

PBL is an uncommon lymphoma with varied clinical and pathologic appearance across ethnic and geographic groups. Due to its low incidence and prevalence, the literature lacks significant knowledge about its clinical behavior and aspects. No analogous incidence of PBL has been reported in Saudi Arabia till now and we provide the first instance in an immunocompetent person in the country. The primary purpose of this paper is to describe the unusual clinicopathologic features of PBL in Saudi Arabia in contrast to previously described cases in the literature.

Regarding clinical presentation, it has been reported that both sinuses, oral, and nasal cavities are the most frequently involved sites in non-HIV patients [5], although has been reported in other sites, including the gastrointestinal tract, skin, and other extra-nodal sites [5]. To emphasize, this patient presented with a soft-tissue mass of the lower extremity. Hence, this raises the possibility of a different spectrum of organ involvement in immunocompetent PBL [6].

Regarding the age distribution, it has been estimated that 64% of patients with non-HIV PBL were over the age of 60 years and 43% were between the ages of 30 and 60 years [5]. This suggests that old age might play a role in the development of PBL secondary to age-related decreased immune function.

Regarding etiology, a considerable percentage of the reported cases of HIV-negative PBL are noticed to have immunosuppression. For example, a systemic review of HIV-negative PBL reported that 58% of cases were EBV positive and 7% were human herpesvirus-8 (HHV-8) positive, concluding that the EBV virus may play a significant role in the development of PBL in HIV-negative patients whereas HHV-8 plays a negligible role [5]. In addition, immunosuppression secondary to post-transplantation, immune-related disease, and current or previous malignancy contributed to the development of PBL in 21% of reported cases [5]. In contrast, our patient had no factors suggestive of immunosuppression. Thus, it raises the possibility of factors other than HIV or immunosuppression that play an important role in the development of PBL in immunocompetent individuals.

Concerning the pathological characteristics of PBL in immunocompetent individuals, it appears that it has very complex cytomorphologic and immunophenotypic characteristics with wide differential diagnosis. PBL expresses immunoreactivity for plasma cell markers (CD38, CD138) and is weakly positive or negative for B-cell markers CD45 and CD20. CD79a is positive in approximately 50-85% of all PBL cases [7].

For instance, in this case, a core biopsy from the thigh was received in formalin and stained with hematoxylin and eosin stain. It consisted of multiple cores, with the longest being 1.5 cm in maximum dimension. The tissue was mostly necrotic showing perivascular viable aggregates of pleomorphic malignant cells. An immunohistochemical study revealed focal CD45 positivity, diffuse CD45Ro, CD30, CD43, MUM-1, and BCL-2. Scattered cells with CD79 were identified. However, it was negative for CD2, CD3, CD4 CD5, CD7, CD8, CD10, CD15, CD19, CD20, CD99, Cyclin-D1, BCL-6, PAX-5 ALK-1, TDT, Granzyme-B, EMA, CK, S100, and Synaptophysin. Proliferative marker Ki67 was high at about 80%. A diagnosis of nalignant hematopoietic neoplasm was rendered. The differential diagnosis included granulocytic sarcoma and anaplastic lymphoma. The tissue at this stage was exhausted, and fine-needle aspiration from the thigh was attempted. A sample was then sent for flow cytometry study and another was processed in the Sure path machine to get cyto-spin material on which further immunohistochemical study was performed using CD31, CD56, CD68, CD99, CD117, CD138, CD168, and MPO antibodies. The only positive and strong reaction was for MPO, and other antibodies showed negative reactions favoring a diagnosis of granulocytic sarcoma. The immunophenotyping of the fine-needle aspiration by flow cytometry showed a prominent mature B-cell population of about 38% of the leukocytes analyzed. They were positive for CD19, CD33, CD71, CD79A, CD38, CD138, partial 36% HLA-DR, and surface kappa light chain restriction. However, they were negative for TDT, MPO, CD56, CD20, CD10, and CD5. These cells showed medium forward scatter and side scatter (large and vacuolated). This raised the possibility of plasmablastic lymphoma, especially because the smear showed that the malignant cells exhibited eccentric nuclei and some with perinuclear Hoff. The fine-needle aspiration was repeated, and the immunohistochemical study was done on the cell block. It was negative for CD19, CD20, CD31, CD34, CD68, CD99, and CD117 antibodies. Surprisingly, it was negative for MPO too, while CD138 and EBV-in situ hybridization were positive confirming the diagnosis of PBM. Hence, cell block was more reliable than the cyto-spin material for immunohistochemical study. In addition, correlation with the flow cytometry immunophenotyping is an important tool to reach the definitive and correct diagnosis of hematopoietic neoplasms [8].

Considering the management of PBL, there is no optimal chemotherapy protocol for the treatment [9]. Cyclophosphamide, doxorubicin, vincristine, and prednisone (CHOP) used to be the standard therapeutic chemotherapy regimen but a more intensive regimen has been recommended by the National Comprehensive Cancer Network [10] such as cyclophosphamide, vincristine, doxorubicin, high-dose methotrexate/ifosfamide, etoposide, and high-dose cytarabine (CODOX-M/IVAC); infusional etoposide, vincristine and doxorubicin with bolus cyclophosphamide and prednisone (infusional EPOCH); and hyperfractionated cyclophosphamide, vincristine, doxorubicin, and dexamethasone alternating with methotrexate and cytarabine (hyper-CVAD). Despite this, recently, two studies of patients with PBL treated with chemotherapy regimens more intensive than CHOP did not confer a statistical difference in survival [11,12].

Generally, PBL has an unfavorable prognosis and aggressive clinical behavior; however, HIV-positive status is associated with a better response to chemotherapy and longer survival [1,5] of nine months versus 14 months in HIV-negative and HIV-positive patients, respectively [4]. The present patient was negative for HIV, and his PBL occurring in an extra-oral site in addition to the negative expression for CD20 altogether pointed toward a more aggressive course and poorer outcome [13].

## Conclusions

We report a unique extra-oral manifestation of PBL in an immunocompetent patient. The data suggest that PBL should be included in the differential diagnosis of fast-growing soft-tissue masses in immunocompetent middle-aged adults to allow for earlier discovery and treatment, perhaps resulting in a better prognosis.

We recommend that a study be conducted that includes unreported cases of PBL in immunocompetent patients to gain a thorough understanding of the clinicopathological behavior of this malignancy in the Saudi population to facilitate a better management approach and the possibility of improving outcomes.
